# Identification of the powdery mildew resistance gene in wheat breeding line Yannong 99102-06188 *via* bulked segregant exome capture sequencing

**DOI:** 10.3389/fpls.2022.1005627

**Published:** 2022-09-06

**Authors:** Yanjun Mu, Wenping Gong, Yanmin Qie, Xueqing Liu, Linzhi Li, Nina Sun, Wei Liu, Jun Guo, Ran Han, Ziyang Yu, Luning Xiao, Fuyu Su, Wenjing Zhang, Jiangchun Wang, Guohao Han, Pengtao Ma

**Affiliations:** ^1^College of Life Sciences, Yantai University, Yantai, China; ^2^Crop Research Institute, Shandong Academy of Agricultural Sciences, Jinan, China; ^3^Institute of Cereal and Oil Crops, Hebei Academy of Agricultural and Forestry Sciences/Hebei Laboratory of Crop Genetic and Breeding, Shijiazhuang, China; ^4^Institute of Grain and Oil Crops, Yantai Academy of Agricultural Sciences, Yantai, China; ^5^Center for Agricultural Resources Research, Institute of Genetics and Developmental Biology, Chinese Academy of Sciences, Shijiazhuang, China

**Keywords:** wheat powdery mildew, *pmYN99102*, MAS, BSE-Seq, molecular mapping

## Abstract

Powdery mildew of wheat (*Triticum aestivum*), caused by *Blumeria graminis* f.sp. *tritici* (*Bgt*), is a destructive disease that seriously threatens the yield and quality of its host. Identifying resistance genes is the most attractive and effective strategy for developing disease-resistant cultivars and controlling this disease. In this study, a wheat breeding line Yannong 99102-06188 (YN99102), an elite derivative line from the same breeding process as the famous wheat cultivar Yannong 999, showed high resistance to powdery mildew at the whole growth stages. Genetic analysis was carried out using *Bgt* isolate E09 and a population of YN99102 crossed with a susceptible parent Jinhe 13–205 (JH13–205). The result indicated that a single recessive gene, tentatively designated *pmYN99102*, conferred seedling resistance to the *Bgt* isolate E09. Using bulked segregant exome capture sequencing (BSE-Seq), *pmYN99102* was physically located to a ~33.7 Mb (691.0–724.7 Mb) interval on the chromosome arm 2BL, and this interval was further locked in a 1.5 cM genetic interval using molecular markers, which was aligned to a 9.0 Mb physical interval (699.2–708.2 Mb). Based on the analysis of physical location, origin, resistant spectrum, and inherited pattern, *pmYN99102* differed from those of the reported powdery mildew (*Pm*) resistance genes on 2BL, suggesting *pmYN99102* is most likely a new *Pm* gene/allele in the targeted interval. To transfer *pmYN99102* to different genetic backgrounds using marker-assisted selection (MAS), 18 closely linked markers were tested for their availability in different genetic backgrounds for MAS, and all markers expect for *YTU103-97* can be used in MAS for tracking *pmYN99102* when it transferred into those susceptible cultivars.

## Introduction

Common wheat (*Triticum aestivum*) is one of the most important crops to food security for it provides ~20% of the calories consumed by humans (Isham et al., 2021). With an estimated global population of more than nine billion by 2050, wheat production is needed an ~70% growth to meet the food demands (International Wheat Genome Sequencing Consortium (IWGSC), 2014). However, powdery mildew, a global devastating wheat disease caused by *Blumeria graminis* f. sp. *tritici* (*Bgt*), can significantly reduce wheat yield and affect flour quality (Li et al., 2019; Wang et al., 2021). It typically decreases wheat yield by 10–15% and up to 62% in severe cases (Singh et al., 2016).

To control this disease, utilization of host resistance is regarded as the most effective, and environmentally friendly way (Chen, 2013; Ma et al., 2015). Nevertheless, it cannot be ignored that *Bgt* isolates have complex and highly variable virulence structures, so their frequent evolution will lead to the continuous breakdown of resistance genes, particularly in the areas where race-specific resistance genes were widely used. A well-known example was the “boom-bust” of *Pm8*, which led to severe epidemics after extended periods of use in the main wheat production regions of China (He et al., 2011, 2015; An et al., 2019). On the other hand, despite more than 80 formally designated *Pm* genes (*Pm1*-*Pm68*, noting that *Pm8*=*Pm17, Pm18*=*Pm1c, Pm22*=*Pm1e, Pm23*=*Pm4c*, and *Pm31*=*Pm21*) having been reported (Li H. H. et al., 2020; McIntosh et al., 2020; He et al., 2021), most of them cannot be directly applied in wheat production due to undesirable linkage drag, such as a broad-spectrum gene *Pm16* which caused up to 15% yield loss when introduced into wheat backgrounds (Summers and Brown, 2013; Tan et al., 2018). In the modern wheat breeding programs of China, only a few *Pm* genes including *Pm2, Pm4, Pm5, Pm8*, and *Pm21* have been extensively applied in wheat improvement (Jia et al., 2020; Jin et al., 2021), making them face huge selective pressure. Therefore, unceasingly exploring and utilizing the novel *Pm* genes/alleles that could balance the broad resistance and comprehensive agronomic performance is an ongoing and essential process.

Once the effective gene was identified, its accurate and rapid transfer or pyramiding is the key point in breeding practice. In comparison to conventional breeding based on phenotypic selection, marker-assisted selection (MAS) is more effective because it combines both genotypic and phenotypic identification. Using the tightly linked or diagnostic markers, the targeted genes could be selected or excluded in fewer generations and thus promote the breeding process (Jiang et al., 2016). Therefore, the isolation of target genes/loci and the development of their tightly linked markers are the two key factors for MAS. Recent advances in the whole-genome sequencing of wheat and corresponding high-throughput sequencing techniques have significantly accelerated the identification and isolation of the resistance genes (Zhu et al., 2020; Ma et al., 2021). A newly-developed strategy bulked segregant exome capture sequencing (BSE-Seq), which combines bulked segregant analysis (BSA) and the exome sequence strategy, has great potential to accelerate gene mapping, particularly in polyploid species with large and complex genome properties such as common wheat. BSE-Seq could effectively identify the linked interval which is not limited to the multiple gene copies, high similarity among the homoeologs, and various types of mapping segregant populations. More importantly, most of the variations obtained from BSE-Seq are existed in the coding regions, making it an economical but effective method for constructing linkage maps and also analyzing the differentially expressed genes associated with the targeted traits (Dong et al., 2020).

Wheat genotypes carrying high-resistance genes but with poor agronomic performance will be greatly limited in breeding because of multigeneration of backcrossing needed, which is not preferred by breeders (Summers and Brown, 2013; Yu et al., 2022). Genes identified in wheat cultivars/breeding lines can be more easily applied in breeding practice compared with those originated from wheat relatives or landraces (Xu et al., 2015). In this case, wheat breeding lines are of high breeding priority as the donor to improve powdery mildew resistance. Yannong 99102-06188 (YN99102), is an elite wheat breeding line developed by the Yantai Academy of Agricultural Sciences (Yantai, China). It exhibited both high resistance to powdery mildew and elite agronomic traits for consecutive years of observation in the field. To better clarify and use the powdery mildew resistance in YN99102, the objectives of this study were to (i) assess the powdery mildew resistance of YN99102 and determine its inheritance; (ii) rapidly map the *Pm* gene(s) using BSE-Seq; (iii) evaluate and develop the tightly linked markers suitable for MAS.

## Materials and methods

### Plant materials

Wheat breeding line YN99102, a derivative line from the same breeding process as the elite wheat cultivar Yannong 999, was derived from the multiple crosses of Lumai 14 and Lin 9,511 through space mutation breeding (Supplementary Figure 1). YN99102 showed high resistance to powdery mildew at both seedling and adult plant stages. To determine the genetic analysis and map the *Pm* gene(s) at the seedling stage in YN99102, the wheat line Jinhe 13–205 (JH13–205) was used as the susceptible parent to cross with YN99102 to generate F_1_ hybrids, F_2_ populations, and F_2:3_ families. Wheat cultivar Mingxian 169 without any known *Pm* gene, was used as the susceptible control for phenotypic evaluation and served as the *Bgt* inoculum spreader. Eight wheat genotypes with known *Pm* genes on chromosome 2BL, Coker 747 (with *Pm6*) (Wan et al., 2020), Am9/3 (with *Pm33*) (Zhu et al., 2005), CH7086 (with *Pm51*) (Zhan et al., 2014), Liangxing 99 (with *Pm52*) (Zhao et al., 2013), WE35 (with *Pm64*) (Zhan et al., 2014), LS5082 (with *PmLS5082*) (Wu et al., 2019), KN0816 (with *PmKN0816*) (Wang et al., 2021) and Qingxinmai (with *PmQ*) (Li Y. H. et al., 2020) were tested with different *Bgt* isolates in order to compare their reactions with that of YN99102 (Table 1). Forty-two susceptible wheat cultivars from different regions of China were used to evaluate the usefulness of the closely linked markers for MAS of the *Pm* gene(s) in YN99102 (Supplementary Table 1).

**Table 1:** Comparative responses of Yannong 99102-06188 and wheat genotypes with known powdery mildew resistance genes on chromosome arm 2BL to 11 isolates of *Blumeria graminis* f. sp. *tritici* (*Bgt*) with different virulence.

**Genotypes**	***Pm* gene**	**A3**	**A10**	**E05**	**E09**	**E18**	**E20**	**E21**	**E23-1**	**E32**	**E23**	**E31**
Yannong 99102-06188	*pmYN99102*	3	1	1	0	1	0	2	0	0	0	0
Jinhe 13–205	–	4	4	4	4	4	4	4	4	4	4	4
Coker747	*Pm6*	0	0	0	3	3	0	3	0	0	0	2
Am9/3	*Pm33*	4	0	0	0	3	0	2	0	0	0	4
CH7086	*Pm51*	0	0	0	0	0	0	4	0	0	0	0
Liangxing 99	*Pm52*	3	0	0	0	0	0	0	0	0	0	0
WE35	*Pm64*	0	0	0	0	0	0	0	4	0	0	0
LS5082	*PmLS5082*	3	0*	0	0	3	0	0	0	0	0	0
KN0816	*PmKN0816*	0	0	0	0	0	0	0	0	0	0	0
Qingxinmai	*PmQ*	0	4	4	3	3	4	0	4	0	4	3

0–4 scale was used to scored the infection types: 0, 0; 1 and 2 were regarded as resistant phenotypes and 3 and 4 were susceptible phenotypes.

### Phenotypic assessment of reactions to powdery mildew

At the adult stage, YN99102 was inoculated with a mixture of 11 *Bgt* isolates including A3, A10, E05, E09, E18, E20, E21, E23–1, E32, E23, and E31 in the field nurseries with three replicates. The assessments were performed from 2018 to 2021 at Yantai University, Yantai City, Shandong Province, China (121.39'E, 37.52'N). For each replicate, YN99102 was planted with 30 seeds per row in four 1.2-m rows, with Mingxian 169 planted on each side of YN99102 as susceptible control and inoculum spreader. When Mingxian 169 showed severe disease symptoms, the disease reaction of YN99102 was assessed using a 0–9 scale for infection types (ITs), in which 0–4 were considered resistant and 5–9 were susceptible (Sheng and Duan, 1991). Each plant was assessed twice for confirmation.

To determine the inheritance of the powdery mildew resistance in YN99102 at the seedling stage, the *Bgt* isolate E09, which is prevalent in the main wheat producing regions of North China (Zhou et al., 2005), was used to inoculate YN99102, susceptible parent JH13–205, and their F_1_ hybrids, F_2_ population, and F_2:3_ families at the one-leaf stage. Each of the F_2:3_ families was tested with 30 seeds to confirm the phenotypic reaction of the F_2_ plants. The resistance assessment was carried out in a greenhouse in a high humidity environment with a daily cycle of 14 h of light at 22°C and 10 h of darkness at 18°C. The tested seeds were planted in rectangular trays (54 × 28 × 4.2 cm) with 128 wells (3.2 × 3.2 × 4.2 cm) and then inoculated at one leaf stage by dusting the fresh conidia of *Bgt* isolate E09, and Mingxian 169 was planted randomly in the trays as the susceptible control. When the pustules were fully developed on the first leaf of Mingxian 169 about 14–15 days after inoculation, each plant was assessed on a 0–4 scale, plants with ITs 0–2 were regarded as resistant and those with ITs 3 and 4 as susceptible (Sheng, 1988; Wang et al., 2005). Goodness-of-fit was analyzed using the chi-square (χ^2^) test to investigate deviations of the observed phenotypic data of F_2_ populations and F_2:3_ families from theoretically expected segregation ratios.

### BSE-Seq

BSE-Seq was used to rapidly located *Pm* gene(s) in the targeted interval by Oebiotech (Shanghai, China). After the susceptible control Mingxian 169 showed serious powdery mildew symptoms on the first leaf, equal leaf tissues from 30 homozygous resistant and 30 homozygous susceptible F_2:3_ plants of YN99102 × JH13–205, respectively, were randomly collected to construct resistant and susceptible bulks. These two DNA bulks were subjected to exome capture sequencing with deep coverage (~70 ×). The construction, assessment, and sequencing of the libraries were performed as described by (Dong et al., 2020).

Raw sequence reads were filtered using Fastp (v0.12.4) to remove the low-quality reads and adapters used. The high-quality reads were then aligned to IWGSC RefSeq v1.0 genome. After that, raw cohort vcf was worked out with GATK (v4.0.10.1) (McCormick et al., 2015). The minimum-mapping-quality parameter was set as 30 for only high-quality alignment reads used to call variants. SNP calling and density analysis were carried out using sliding window calculation based on the reference of Takagi et al. (2013) The data filtering parameters were set as AF (Allele Frequence) <0.3 or >0.7. Bcftools (v1.9) (Narasimhan et al., 2016) was performed for variants quality filtering with “QUAL > 30” and “DP ≥5.” The statistical model varBScore was carried out to determine the candidate interval. SnpEff (v4.3T) (Cingolani et al., 2012) was used to generate customized databases containing IWGSC v1.1 HC/LC genes for the annotation of the variants.

### Molecular markers analysis

Based on the candidate interval obtained from BSE-Seq, 98 molecular markers linked to the known *Pm* genes in the candidate interval were firstly used to test for polymorphisms between resistant and susceptible parents and bulks (Table 2). Then, the polymorphic markers between the parents and the bulks were used to genotype the F_2:3_ families of YN99102 × JH13–205 for a preliminary mapping of the *Pm* gene(s) in YN99102. Moreover, 70 new markers in the target interval were developed based on the simple sequence repeat (SSR) and small insertion-deletion (InDel) that were discovered by BSE-Seq (Supplementary Table 2).

**Table 2:** Polymorphic and linkage analyses of the markers linked to the powdery mildew resistance genes located on chromosome arm 2BL using the mapping population derived from the cross of Yannong 99102-06188 × Jinhe 13–205.

**Marker**	**Resistance genes**	**Physical location** **(Mb)**	**Polymorphism**		**Linkage to *pmYN99102***	**References**
			**Parents**	**F_2:3_ bulks**		
*CIT02g-1*	*Pm6*	711.0	*-*	*-*	*-*	Wan et al., 2020
*CIT02g-2*	*Pm6*	722.1	+	+	+	Wan et al., 2020
*CIT02g-3*	*Pm6*	699.2	+	+	+	Wan et al., 2020
*CIT02g-4*	*Pm6*	730.2	*-*	*-*	*-*	Wan et al., 2020
*CIT02g-5*	*Pm6*	724.8	*-*	*-*	*-*	Wan et al., 2020
*CIT02g-6*	*Pm6*	694.1	*-*	*-*	*-*	Wan et al., 2020
*CIT02g-7*	*Pm6*	694.1	*-*	*-*	*-*	Wan et al., 2020
*CIT02g-8*	*Pm6*	710.3	*-*	*-*	*-*	Wan et al., 2020
*CIT02g-9*	*Pm6*	710.3	*-*	*-*	*-*	Wan et al., 2020
*CIT02g-10*	*Pm6*	722.1	*-*	*-*	*-*	Wan et al., 2020
*CIT02g-11*	*Pm6*	709.1	*-*	*-*	*-*	Wan et al., 2020
*CIT02g-12*	*Pm6*	722.3	*-*	*-*	*-*	Wan et al., 2020
*CIT02g-13*	*Pm6*	708.2	+	+	+	Wan et al., 2020
*CIT02g-14*	*Pm6*	694.1	*-*	*-*	*-*	Wan et al., 2020
*CIT02g-15*	*Pm6*	722.1	+	+	+	Wan et al., 2020
*CIT02g-16*	*Pm6*	709.8	*-*	*-*	*-*	Wan et al., 2020
*CIT02g-17*	*Pm6*	697.7	+	+	+	Wan et al., 2020
*CIT02g-18*	*Pm6*	698.3	+	+	+	Wan et al., 2020
*CIT02g-19*	*Pm6*	731.0	*-*	*-*	*-*	Wan et al., 2020
*CIT02g-20*	*Pm6*	699.2	+	+	+	Wan et al., 2020
*CIT02g-21*	*Pm6*	730.9	*-*	*-*	*-*	Wan et al., 2020
*CIT02g-22*	*Pm6*	715.6	*-*	*-*	*-*	Wan et al., 2020
*CISSR02g-1*	*Pm6*	704.2	*-*	*-*	*-*	Wan et al., 2020
*CISSR02g-2*	*Pm6*	701.8	*-*	*-*	*-*	Wan et al., 2020
*CISSR02g-3*	*Pm6*	701.1	*-*	*-*	*-*	Wan et al., 2020
*CISSR02g-5*	*Pm6*	699.1	*-*	*-*	*-*	Wan et al., 2020
*CISSR02g-6*	*Pm6*	700.4	+	+	+	Wan et al., 2020
*CINAU117*	*Pm6*	614.9	*-*	*-*	*-*	Qin et al., 2011
*CINAU118*	*Pm6*	*-*	*-*	*-*	*-*	Qin et al., 2011
*CINAU119*	*Pm6*	*-*	*-*	*-*	*-*	Qin et al., 2011
*CINAU120*	*Pm6*	*-*	*-*	*-*	*-*	Qin et al., 2011
*CINAU121*	*Pm6*	*-*	*-*	*-*	*-*	Qin et al., 2011
*CINAU122*	*Pm6*	*-*	*-*	*-*	*-*	Qin et al., 2011
*CINAU125*	*Pm6*	653.3	*-*	*-*	*-*	Qin et al., 2011
*CINAU126*	*Pm6*	*-*	*-*	*-*	*-*	Qin et al., 2011
*CINAU127*	*Pm6*	677.3	*-*	*-*	*-*	Qin et al., 2011
*CINAU128*	*Pm6*	-	*-*	*-*	*-*	Qin et al., 2011
*CINAU129*	*Pm6*	-	*-*	*-*	*-*	Qin et al., 2011
*CINAU131*	*Pm6*	689.1	*-*	*-*	*-*	Qin et al., 2011
*CINAU132*	*Pm6*	-	*-*	*-*	*-*	Qin et al., 2011
*CINAU133*	*Pm6*	*-*	*-*	*-*	*-*	Qin et al., 2011
*CINAU134*	*Pm6*	*-*	*-*	*-*	*-*	Qin et al., 2011
*CINAU135*	*Pm6*	690.2	*-*	*-*	*-*	Qin et al., 2011
*CINAU136*	*Pm6*	695.8	*-*	*-*	*-*	Qin et al., 2011
*CINAU137*	*Pm6*	*-*	*-*	*-*	*-*	Qin et al., 2011
*CINAU138*	*Pm6*	*-*	*-*	*-*	*-*	Qin et al., 2011
*CINAU139*	*Pm6*	753.0	*-*	*-*	*-*	Qin et al., 2011
*CINAU140*	*Pm6*	747.2	*-*	*-*	*-*	Qin et al., 2011
*CINAU141*	*Pm6*	710.9	*-*	*-*	*-*	Qin et al., 2011
*CINAU142*	*Pm6*	723.0	*-*	*-*	*-*	Qin et al., 2011
*CINAU143*	*Pm6*	715.0	*-*	*-*	*-*	Qin et al., 2011
*CINAU144*	*Pm6*	710.9	*-*	*-*	*-*	Qin et al., 2011
*NAU/STSBCD135-2*	*Pm6*	738.6	-	-	-	Qin et al., 2011; Tan et al., 2018
*CINAU123*	*Pm6*	*-*	*-*	*-*	*-*	Qin et al., 2011
*CINAU124*	*Pm6*	*-*	*-*	*-*	*-*	Qin et al., 2011
*Xicscl172*	*Pm52*	595.7	+	+	-	Wu et al., 2019
*Xicscl174*	*Pm52*	595.7	+	+	-	Wu et al., 2019
*Xicsl326*	*Pm52*	581.0	+	+	-	Wu et al., 2019
*Xicscl795*	*Pm52*	585.0	*-*	*-*	-	Wu et al., 2019
*Xicsl163*	*Pm52*	596.4	*-*	*-*	-	Wu et al., 2019
*Xicsl224*	*Pm52*	556.6	*-*	*-*	-	Wu et al., 2019
*Xicsl275*	*Pm52*	382.9	*-*	*-*	-	Wu et al., 2019
*Xicsl306*	*Pm52*	607.2	*-*	*-*	-	Wu et al., 2019
*Xicsl34*	*Pm52*	564.8	*-*	*-*	-	Wu et al., 2019
*Xicsl62*	*Pm52*	556.6	*-*	*-*	-	Wu et al., 2019
*Xicsl90*	*Pm52*	603.6	*-*	*-*	-	Wu et al., 2019
*Xicsl234*	*Pm52*	596.6	+	+	-	Wu et al., 2019
*Xgwm120*	*Pm52*	615.7	*-*	*-*	-	Zhao et al., 2013
*Xwmc175*	*Pm52, Pm63*	670.6	*-*	*-*	-	Zhao et al., 2013; Tan et al., 2018
*Xgwm120*	*Pm52, Pm63*	615.8	*-*	*-*	-	Zhao et al., 2013; Tan et al., 2018
*Xwmc441*	*Pm52, Pm63*	598.0	*-*	*-*	-	Zhao et al., 2013; Tan et al., 2018
*WGGBH1212*	*Pm64*	656.6	*-*	*-*	-	Zhang et al., 2019
*WGGBH1260*	*Pm64*	695.1	*-*	*-*	-	Zhang et al., 2019
*WGGBH134*	*Pm64*	670.6	*-*	*-*	-	Zhang et al., 2019
*WGGBH1364*	*Pm64*	695.4	*-*	*-*	-	Zhang et al., 2019
*WGGBH218*	*Pm64*	699.2	*-*	*-*	-	Zhang et al., 2019
*WGGBH252*	*Pm64*	732.3	*-*	*-*	-	Zhang et al., 2019
*WGGBH612-5*	*Pm64*	710.3	*-*	*-*	-	Zhang et al., 2019
*WGGBH686*	*Pm64*	680.0	*-*	*-*	-	Zhang et al., 2019
*WGGBH913*	*Pm64*	715.0	*-*	*-*	-	Zhang et al., 2019
*WGGBH1099*	*Pm64*	705.5	*-*	*-*	-	Zhang et al., 2019
*stars419*	*Pm63*	710.3	*-*	*-*	-	Tan et al., 2019
*Xbcd135-2*	*Pm63*	723.4	*-*	*-*	-	Tan et al., 2019
*BE405017*	*Pm51*	767.1	*-*	*-*	-	Zhan et al., 2014
*BE444894*	*Pm51*	765.3	*-*	*-*	-	Zhan et al., 2014
*BQ246670*	*Pm51*	709.8	*-*	*-*	-	Zhan et al., 2014
*Cos66*	*Pm51*	747.2	*-*	*-*	-	Zhan et al., 2014
*Xbarc159*	*Pm51*	793.0	*-*	*-*	-	Zhan et al., 2014
*Xwmc332*	*Pm51, Pm63*	739.4	*-*	*-*	-	Zhan et al., 2014; Tan et al., 2018
*Xwmc332*	*Pm51, Pm63*	739.4	*-*	*-*	-	Zhan et al., 2014; Tan et al., 2018
*Xgwm526*	*Pm33*	774.1	*-*	*-*	-	Zhu et al., 2005
*Xwmc317*	*Pm33*	784.3	*-*	*-*	-	Zhu et al., 2005
*Xicsq10*	*PmQ*	750.0	*-*	*-*	-	Li H. H. et al., 2020
*Xicsq129*	*PmQ*	740.3	*-*	*-*	-	Li Y. H. et al., 2020
*Xicsq253*	*PmQ*	730.6	*-*	*-*	-	Li H. H. et al., 2020
*Xicsq347*	*PmQ*	720.9	*-*	*-*	-	Li H. H. et al., 2020
*Xicsq405*	*PmQ*	710.7	*-*	*-*	-	Li H. H. et al., 2020
*Xicsq453*	*PmQ*	730.8	*-*	*-*	-	Li Y. H. et al., 2020

In the polymorphism column, “+” represents polymorphic or linked, and “–” represents non-polymorphic or unlinked, and in physical location, “–” represents no data.

PCR amplification was performed as described by Han et al. (2022a,b) with minor modification. The PCR products were then separated in 8% non-denaturing polyacrylamide gels with a 29:1 ratio of acrylamide and bisacrylamide, finally visualized by silver staining.

### Map construction and functional annotation

After obtaining phenotyping data from the evaluation of disease resistance and the genotyping data of the F_2:3_ families from molecular marker analysis, the linkage map of the *Pm* gene in YN99102 was constructed using MAPMAKER 3.0 (Lincoln et al., 1992) and the Kosambi function as reported previously (Kosambi, 1944). Functional annotation was performed based on the information from IWGSC RefSeq [version 1.0; The International Wheat Genome Sequencing Consortium (IWGSC, 2018)].

### Comparison with the known *Pm* genes on the chromosome arm 2BL

Considering that the *Pm* gene in YN99102 was assigned to the chromosome 2BL, YN99102 and eight wheat genotypes also carrying known *Pm* genes on chromosome 2BL, including Coker 747 (with *Pm6*), Am9/3 (with *Pm33*), CH7086 (with *Pm51*), Liangxing 99 (with *Pm52*), WE35 (with *Pm64*), LS5082 (with *PmLS5082*), KN0816 (with *PmKN0816*) and Qingxinmai (with *PmQ*), were tested against 11 *Bgt* isolates that were collected from the diseased wheat fields in different wheat growing areas of China to compare their resistance spectrum (Table 1). Each isolate was developed through single-spore purification and separately stored in glass tubes with three layers of gauzes. The methods of inoculation and incubated conditions were described previously (Wu et al., 2019).

To further distinguish the *pmYN99102* from the documented *Pm* genes on chromosome arm 2BL at the level of genetic diversity, 98 markers closely linked to those *Pm* genes were tested for polymorphisms between resistant and susceptible parents and bulks derived from the F_2:3_ families of YN99102 × JH13–205 to investigate the genetic diversity of the candidate interval of *Pm* gene in YN99102 and the known *Pm* genes in chromosome arm 2BL (Table 2).

### Evaluation of the closely linked markers for MAS

To evaluate the applicability of the markers for MAS breeding, 42 susceptible wheat cultivars from different regions of China were tested with the closely linked or co-segregated markers. The markers which were able to consistently amplify polymorphic band(s) between YN99102 and these susceptible cultivars were regarded as effective for MAS in those genetic backgrounds (Supplementary Table 1). To transfer the *Pm* gene(s) in YN99102 to applicable backgrounds, these cultivars were crossed with YN99102 to construct BC_1_F_2_ and F_3_ segregation populations for MAS.

## Results

### Evaluation and inheritance of powdery mildew resistance in YN99102

For the adult plant investigations with powdery mildew in the field, YN99102 showed high resistance with ITs 0–1 to the *Bgt* mixture including *Bgt* isolates A3, A10, E05, E09, E18, E20, E21, E23–1, E32, E23 and E31 over the consecutive growing seasons from 2018 to 2021.

Then, the *Bgt* isolate E09 was used to determine the inheritance of powdery mildew resistance in YN99102 at the seedling stage. When inoculated with this isolate, YN99102 was highly resistant with IT 0, whereas JH13–205 was highly susceptible with IT 4. All the 10 F_1_ plants of the cross YN99102 × JH13–205 were susceptible with IT 4, indicating the resistance of YN99102 to *Bgt* isolate E09 was controlled by recessive *Pm* gene(s). The F_2_ populations segregated in 31 resistant plants scored as IT 0, and 77 susceptible plants scored as IT 4, which fits a theoretical ratio of 1:3 for the monogenic segregation (χ^2^ = 0.79; *P* = 0.37). Subsequently, all the 108 F_2_ plants were transplanted in the field to generate F_2:3_ families for the confirmation of the homozygous or heterozygous genotype of the susceptible F_2_ plants. The 108 F_2:3_ families segregated with 31 homozygous resistant (rr), 52 segregating (Rr), and 25 homozygous susceptible (RR), and the phenotypic result of F_2:3_ families further confirmed the ratio of monogenic inheritance of the powdery mildew resistance 1:2:1 (χ^2^ = 0.48; *P* = 0.49) (Table 3). Therefore, it suggested that the resistance to *Bgt* isolate E09 in YN99102 was controlled by a single recessive gene, tentatively designated as *pmYN99102*.

**Table 3:** Segregation ratios of F_2_ and F_2:3_ generations of Yannong 99102-06188 (YN) and Jinhe 13-205 (JH) following inoculation with *Blumeria graminis* f. sp. *tritici* (*Bgt*) isolate E09 at the seedling stage.

**Parent and cross^a^**	**Generation^b^**	**Observed ratio^c^**	**Expected ratio**	**χ^2^**	***P***
YN	RP	R:S=10:0			
JH	SP	R:S=0:10			
YN × JH F_1_	F_1_	R:S=0:10			
YN × JH F_2_	F_2_	R:S=31:77	1:3	0.79	0.37
YN × JH F_3_	F_2:3_	HR:Seg:HS=31:52:25	1:2:1	0.48	0.49

a YN, Yannong 99102-06188; JH, Jinhe 13–205.

b RP, Resistant parent; SP, Susceptible parent.

c R, Resistant; S, Susceptible; HR, homozygous resistant; Seg, segregating; HS, homozygous susceptible.

### SNP calling and confirmation of candidate interval

**Figure 1:**
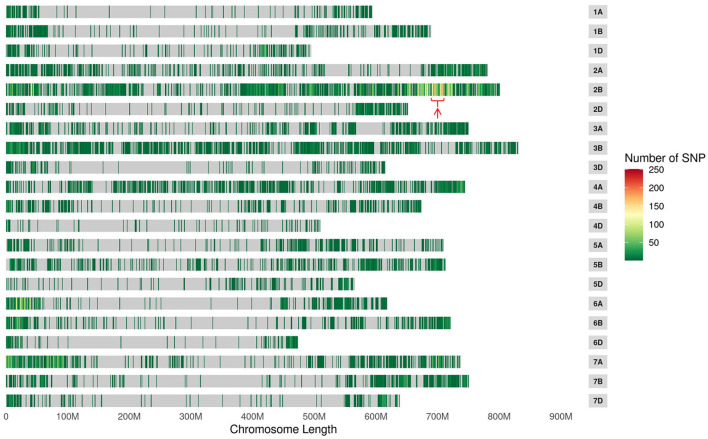
The distribution of single nucleotide polymorphisms (SNPs) and significant candidate intervals (indicated with red arrow) across the 21 wheat chromosomes.

**Figure 2:**
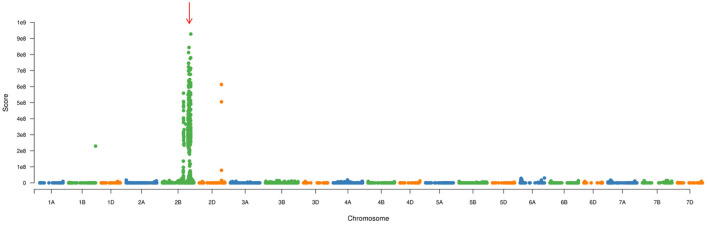
Manhattan plot of the varBScore across the 21 wheat chromosomes for analyzing the candidate region (indicated with red arrow).

To confirm the genetic position of *pmYN99102*, the resistant and susceptible DNA bulks were genotyped using BSE-Seq. Based on the results of BSE-Seq, a total of 32,711 high-quality SNPs were identified between the resistant and susceptible bulks by ΔSNP index analysis, which was distributed on all of the wheat chromosomes (Figure 1). Among them, 10,731 (32.8%) SNPs were detected on chromosome arm 2B, 2,978 (27.8%) SNPs enriched on chromosome arm 2BL 691.0–724.7 Mb with varBScore analysis (Figure 2), indicating that the *pmYN99102* was likely located in this 33.7 Mb interval on chromosome arm 2BL.

### Molecular mapping of *PmYN99102* and prediction of the candidate genes

**Figure 3:**
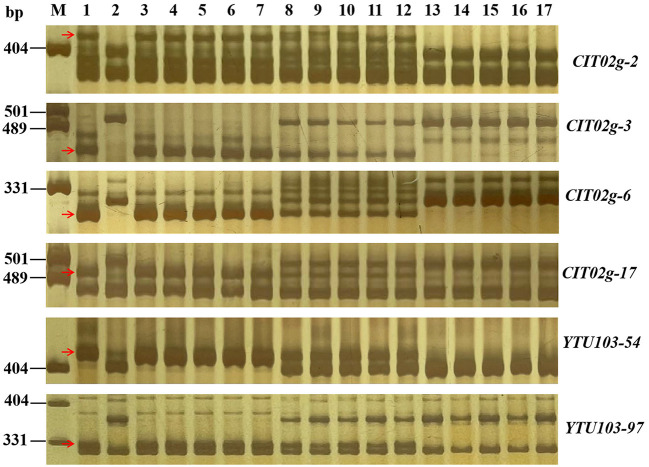
Amplification patterns of *pmYN99102*-linked markers *CIT02g-2* (A), *CIT02g-3* (B), *CIT02g-6* (C), *CIT02g-17* (D), *CIT02g-54* (E) and *YTU103-97* in genotyping resistant parent Yannong 99102-06188 (YN99102), susceptible parent Jinhe13–205 (JH13–205) and randomly selected F_2:3_ families of YN99102 × JH13–205. Lane M, pUC18 *Msp* I; lanes 1–2, YN99102 and JH13–205; lanes 3–7, homozygously resistant F_2:3_ families; lanes 8–12, heterozygously F_2:3_ families; lanes 13–17, homozygously susceptible F_2:3_ families. The red arrows were used to indicate the polymorphic bands linked to *pmYN99102*.

**Figure 4:**
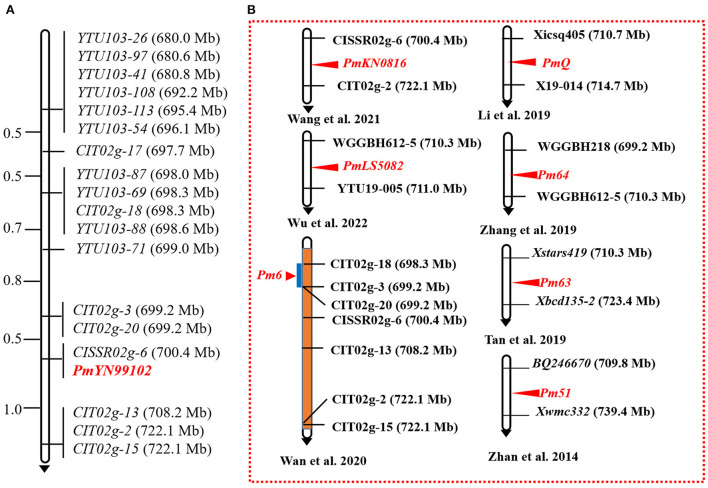
Linkage map of *pmYN99102* using the F_2:3_ families of Yannong 99102-06188 × Jinhe13–205 (A) and the physical locations of cataloged *Pm* genes on chromosome arm 2BL (B). Genetic distances in cM are showed to the left. The black arrows point to the centromere.

With the candidate interval confirmed, 98 previously reported markers linked to the candidate interval on 2BL (Table 2) and 70 newly developed markers based on the results of BSE-Seq (Supplementary Table 2) were used to screen polymorphism between the resistant and susceptible parents and bulks. Among them, 19 markers, including 10 newly developed markers (Table 4), amplified consistent polymorphisms between the resistant and susceptible parents and bulks, which were used to genotype the F_2:3_ families of the cross of YN99102 × JH13–205 to construct the linkage map and further narrow down the candidate interval of *pmYN99102* (Figure 3). The result showed that *pmYN99102* was flanked by markers *CIT02g-3*/*CIT02g-20* and *CIT02g-13*/*CIT02g-2*/*CIT02g-15* with genetic distances of 0.5 and 1.0 cM, corresponding to 699.2–708.2 Mb physical interval, and co-segregated with *CISSR02g-6* (700.4 Mb) according to the IWGSC Chinese Spring reference genome v1.0 (Figure 4). In this interval, we obtained a total of 76 high confidence genes based on the gene annotation results. Among them, four genes *TraesCS2B01G505200, TraesCS2B01G507000, TraesCS2B01G507100* and *TraesCS2B01G509000* were related to disease resistance and were regarded as the candidate genes of *pmYN99102* (Supplementary Table 3).

**Table 4:** *pmYN99102*-linked markers developed by bulked segregant exome capture sequencing (BSE-Seq).

**Marker**	**Location**	**FORWARD PRIMER1 (5'-3')**	**REVERSE PRIMER1 (5^′^−3^′^)**
YTU103-26	chr2B:680042044-680043045	TGTCGCTGTCACTTGCTGAT	TCACCAGCACATGAGTCACC
YTU103-41	chr2B:680805143-680806144	AGCTTGAACTTGCCGGCTAT	CAGCTTCATGGACAGGCTCA
YTU103-54	chr2B:696080372-696081373	AGGGCAAAAGATGGAGGTCG	TCGTTCAAGGGCATCAGCAT
YTU103-69	chr2B:698301932-698302933	CGAGCGTGATGTAGACCTCC	GTTTTTCCAGGCCAGCAAGG
YTU103-71	chr2B:698963503-698964504	CTCGTCGCCAAATGCTGATG	AGGCGGTTGATAGAGCACAC
YTU103-87	chr2B:697993507-697994508	AGCCGTTCCTTGATGTCAGG	ACTCCCATCGAGGATCCACA
YTU103-88	chr2B:698590771-698591772	CTCGCGCAAGAACACACAAA	ACCTGCTCTGGATGCTTGAC
YTU103-97	chr2B:680597479-680598480	CTAGGGCTGGACCAGTTTGG	AGTTGTGGAAATCGGCGGAT
YTU103-108	chr2B:692161592-692162593	GTCAGGCCTGGGAGGAATTC	CCATGGAAGGAGGAGGAGGA
YTU103-113	chr2B:695372385-695373386	CTGCTGACAGTACGGTGTGT	CGCCAGCAGATTAACCATGC

### Comparisons of *PmYN99102* and the known *Pm* genes on chromosome arm 2BL

**Figure 5:**
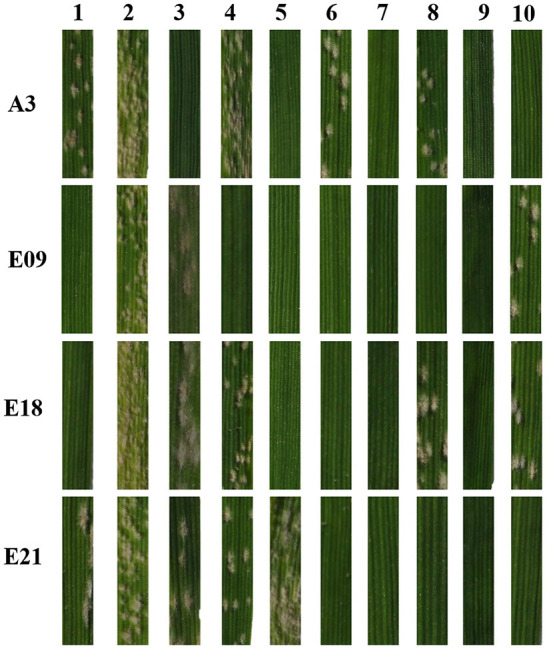
Reaction patterns of wheat breeding line Yannong 99102-06188 (YN99102) and the resistant donors with cataloged *Pm* genes on 2BL chromosome to selected *Blumaria graminis* f. s. *tritici* isolates A3, E09, E18, and E21. 1: YN99102; 2: susceptible parent Jinhe13-205 (JH13–205); 3: Coker747 (*Pm6*); 4: Am9/3 (*Pm33*); 5: CH7086 (*Pm51*); 6: Liangxing 99 (*Pm52*); 7: WE35 (*Pm64*); 8: LS5082 (*PmLS5082*); 9: KN0816 (*PmKN0816*); 10: Qingxinmai (*PmQ*).

To identify the relationship between *pmYN99102* and the known *Pm* genes on chromosome arm 2BL, YN99102 (with *pmYN99102*), Coker747 (with *Pm6*), Am9/3 (with *Pm33*), CH7086 (with *Pm51*), Liangxing99 (with *Pm52*), WE35 (with *Pm64*), LS5082 (with *PmLS5082*), KN0816 (with *PmKN0816*) and Qingxinmai (with *PmQ*) were tested against 11 *Bgt* isolates to evaluate their resistance spectrum. The results showed that YN99102 was resistant to 10 of 11 (90.9%) isolates, while Coker747, Am9/3, CH7086, Liangxing99, WE35, LS5082, KN0816 and Qingxinmai was resistant to 8 of 11 (72.7%), 8 of 11 (72.7%), 10 of 11 (90.9%), 10 of 11 (90.9%), 10 of 11 (90.9%), 9 of 11 (81.8%), 11 of 11 (100%), 3 of 11 (27.2%), respectively (Figure 5 ; Table 1). Even though YN99102, CH7086, and Liangxing 99 were all resistant to 10 out of 11, they showed significantly different phenotypes against different isolates. Therefore, YN99102 had different resistance spectrum from the known *Pm* genes on 2BL.

To further distinguish *pmYN99102* from the known *Pm* genes on 2BL, 98 previously reported markers that were closely linked to the documented *Pm* genes on chromosome arm 2BL were test the polymorphisms between the resistant and susceptible bulks derived from the F_2:3_ families of YN99102 × JH13–205 (Table 2). Among them, 12 markers, including eight markers for *Pm6* (*CIT02g*−*2, CIT02g*−*3, CIT02g*−*13, CIT02g*−*15, CIT02g*−*17, CIT02g*−*18, CIT02g*−*20, CISSR02g*−*2*) and four markers for *Pm52* (*Xicscl172, Xicscl174, Xicsl326*, and *Xicsl234*) amplified polymorphisms between the resistant and susceptible parents and bulks, while other 86 markers showed no polymorphism. Four markers closely linked to *Pm52* were not linked to *pmYN99102* (Table 2). Molecular markers analysis combined with different resistance spectrum demonstrated that *pmYN99102* is most likely different from the known *Pm* genes on chromosome arm 2BL.

### Molecular markers for MAS

**Figure 6:**
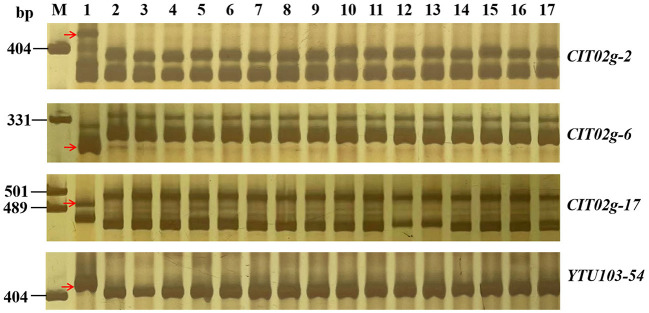
Amplification patterns of *pmYN99102*-linked markers *CIT02g-2* (A) and *CIT02g-6* (B), *CIT02g-17* (C) and *YTU103-54* (D) in Yannong 99102-06188 (YN99102), Jinhe13–205 (JH13–205), and 15 wheat cultivars/breeding lines susceptible to powdery mildew. M, DNA marker pUC18 *Msp* I; lanes 1 and 2, YN99102 and JH13–205; and lanes 3–17: Shannong 1,538, Hanmai 13, Huaimai 0,226, Zhoumai 27, Xinong 979, Lumai 185, Zhongyu 1,311, Jimai 268, Tainong 1,014, Jimai 229, Jimai 21, Jimai 20, Daimai 2,173, Zhoumai 1,751, Jinan 17. The white arrows indicate the polymorphic bands in YN99102.

To better use *pmYN99102* in MAS, 18 markers closely linked to *pmYN99102* were tested for their availability in the 42 susceptible wheat cultivars for MAS (Figure 6; Supplementary Table 1). All markers except for *YTU103–97* could amplify polymorphic bands between YN99102 and most of the 42 susceptible cultivars, suggesting that these markers can be used in MAS for tracking *pmYN99102* when transferred into those cultivars. *YTU103–97* amplified consistent bands in 38 out of 42 susceptible cultivars, meaning that these two markers were less appropriate for MAS of *pmYN99102*.

## Discussion

In the present study, using genetic analysis, BSE-Seq, and molecular marker analysis, we accurately and rapidly identified a recessive *Pm* gene *pmYN99102* in YN99102 and localized it to a 0.9 Mb interval (699.2–708.2 Mb) on chromosome arm 2BL, an enrichment region carrying many *Pm* genes (Wu et al., 2019). Eleven *Pm* genes have been reported to be located on chromosome arm 2BL from various gene donors, including *Pm6* from *Triticum timopheevii* (Wan et al., 2020), *Pm33* from *T. persicum* Vav. (Zhu et al., 2005), *Pm51* from a *Thinopyrum ponticum* introgression line (Zhan et al., 2014), *Pm52* from Chinese wheat cultivar Liangxing 99 (Wu et al., 2019), *Pm63* from Iranian wheat landrace PI 628024 (Tan et al., 2019), *Pm64* from wild emmer (Zhang et al., 2019), *PmQ* from Chinese wheat landrace Qingxinmai (Li H. H. et al., 2020), *MlZec1* and *MlAB10 both from T. dicoccoides* (Mohler et al., 2005; Maxwell et al., 2010), *PmKN0816* from the Chinese wheat breeding line KN0816 (Wang et al., 2021) and *PmLS5082* from the Chinese wheat breeding line LS5082 (Wu et al., 2019), suggesting chromosome arm 2BL is a potential R gene-rich region and complex molecular modules and mechanisms may be involved in the chromosome arm 2BL. Compared with those documented genes, *pmYN99102* (699.2–708.2 Mb) could be clearly distinguished from eight of them: *Pm33* (773.2–784.3 Mb), *Pm51* (709.8–739.4 Mb), *Pm52* (581.0–585.0 Mb), *Pm63* (710.3–723.4 Mb), *PmQ* (710.7–714.7 Mb), *MlZec1* and *MlAB10* (both 796.7–780.0 Mb), and *PmLS5082* (710.3–711.0 Mb) based on their physical locations and/or origins. However, the physical intervals of three genes *Pm6* (698.3–699.2 Mb), *Pm64* (699.2–710.3 Mb), and *PmKN0816* (700.4–710.3 Mb) overlapped that of *pmYN99102* (699.2–708.2 Mb) and hence it is necessary to clarify their relationships.

*T. timopheevii* derived *Pm6* was the first *Pm* gene identified on chromosome 2BL and was transferred into the wheat genetic background in the form of wheat-*T. timopheevii* 2B/2G introgression lines (Jorgensen and Jensen, 1973; Bennett, 1984; Ji et al., 2008). The 2G chromosome introgression segment carrying *Pm6* has strong recombination suppression in the wheat genome, so seven of the eight *Pm6*-linked markers used in this study had no recombination between the 2G chromosome introgression segment carrying *Pm6* and the corresponding wheat segment, while they showed normal recombination frequency as common wheat in the *pmYN99102* interval. Moreover, only 8 of 55 *Pm6*-linked markers showed polymorphisms between YN99102 and JH13–205, and their derivative F_2:3_ resistant and susceptible bulks (Table 2), which revealed a distinct genetic diversity between the intervals of *pmYN99102* and *Pm6*. Meanwhile, YN99106 had a broader resistance spectrum than Coker 747 (with *Pm6*). Critically, YN99106 was resistant to *Bgt* isolate E09 and *pmYN99102* was identified by inoculating the *Bgt* isolate E09, whereas Coker 747 (with *Pm6*) was susceptible to the *Bgt* isolate E09 (Table 1). It was reported that Coker 747 was moderate effectiveness at the one-leaf stage to the two-leaf stage, but showed gradually increased resistance from the third leaf stage and reached complete resistance at the fourth leaf stage and later (Qin et al., 2011). Whereas, YN99102 exhibited high resistance to *Bgt* isolates from the one-leaf stage and continued through all growth stages. Therefore, the combined evidence indicated that *pmYN99102* was different from *Pm6. Pm64*, derived from a wheat-*T. dicoccoides* introgression line showed a different resistance spectrum from *pmYN99102* (Table 1). When tested with 10 *Pm64*-linked markers, none of them amplified polymorphisms in YN99102 and JH03–125 and their mapping population. *PmKN0816* was a broad-spectrum resistance gene and also discovered in a Chinese wheat breeding line, and its donor KN0816 was resistant to all the 11 tested *Bgt* isolates, but YN99102 was susceptible to the *Bgt* isolate A03. In addition, all of the three *Pm* genes *Pm6, Pm64*, and *PmKN0816* followed dominant inheritance pattern, while *pmYN99102* was distinctively recessive. Taken together, *pmYN99102* is most likely a new gene different from the cataloged *Pm* genes on chromosome 2BL based on their origins, chromosome intervals, resistance spectrum, and inheritance pattern. Of course, allelism tests and cloning of these genes are necessary in the future to finally determine their relationships in such a mysterious interval containing multiple and complex resistance genes. Further validation on the four candidate genes of *pmYN99102* that was directly related to disease resistance (Supplementary Table 3) will be our focus in the near future.

When a resistance gene was identified, less linkage drag is the critical factor associated with its easy use in wheat breeding programs, but often, disease resistance is at the expense of some agronomic traits and reduced plant adaptation (Deng et al., 2017; Ma et al., 2018; Han et al., 2022b). Fortunately, YN99102 is an elite derivative line from the same breeding process as a famous wheat cultivar Yannong 999 in China, which is the first wheat cultivar to exceed 800 kilograms per mu yield in China. In the breeding process of Yannong 999, two prominent breeding lines, Yannong 99102-06072 and Yannong 99102-06188 (YN99102) were selected as the candidate lines. Of the two lines, Yannong 99102-06072 has favorable synergy between yield and quality, making it the current Yannong 999 official registration which met the breeding goal of high yield and quality; whereas YN99102 has the advantages of both powdery mildew resistance and high yield, making it a valuable resistance resource for both wheat breeding and genetic study. To facilitate the transfer of *pmYN99102* in MAS, we evaluated the applicability of 17 markers including 10 newly developed markers and seven reported markers in 42 susceptible cultivars (Supplementary Table 1). The results showed that all markers except for *YTU103–97* were polymorphic between YN99102 and most of the 42 susceptible cultivars, suggesting that these markers can be used in MAS for detecting *pmYN99102* once it was introduced to those susceptible cultivars. In fact, we have obtained the BC_1_F_2_ and F_3_ segregation populations from the cross of some applicable cultivars and YN99102 currently. We believe that *pmYN99102* will release its full potential following the selection for resistance and agronomic performance in wheat breeding programs.

## Conclusion

In conclusion, using BSE-Seq and molecular markers, we identified a powdery mildew resistance gene *pmYN99102* in the wheat breeding line YN99102. Based on the analysis of physical location, origin, resistant spectrum, and inherited pattern, *pmYN99102* is most likely a new *Pm* gene. Molecular markers available for marker-assisted selection were also selected for tracking *pmYN99102* in breeding. Our study can be valuable for enhancing the genetic diversity of powdery mildew resistance in breeding.

## Data availability statement

The datasets presented in this study can be found in online repositories. The names of the repository/repositories and accession number(s) can be found in the article/Supplementary material.

## Author contributions

PM, GH, and JW conceived the research. YM, WG, YQ, ZY, LX, FS, and WZ performed the experiments. JW and XL developed the experimental materials. NS and WL performed the phenotypic assessment. GH, JG, and RH analyzed the data. PM wrote the manuscript. All authors read and approved the final manuscript.

## Funding

This research was financially supported by the National Natural Science Foundation of China (32072053, 31971874) and the Key Research and Development Project of Shandong Province (2020CXGC010805, 2021LZGC009).

## Conflict of interest

The authors declare that the research was conducted in the absence of any commercial or financial relationships that could be construed as a potential conflict of interest.

## Publisher's note

All claims expressed in this article are solely those of the authors and do not necessarily represent those of their affiliated organizations, or those of the publisher, the editors and the reviewers. Any product that may be evaluated in this article, or claim that may be made by its manufacturer, is not guaranteed or endorsed by the publisher.

## Supplementary material

Supplementary Figure 1 Genealogy of the wheat breeding line Yannong 99102-06188 and its relationship with the wheat cultivar Yannong 999.

Supplementary Table 1 Validation of *pmYN99102*-linked markers on 42 Chinese wheat cultivars/breeding lines in marker-assisted selection (MAS) breeding.

Supplementary Table 2 Molecular markers developed by bulked segregant exome capture sequencing (BSE-Seq).

Supplementary Table 3 Gene annotation in the candidate interval of wheat powdery mildew resistance gene *pmYN99102*.
