# Supplementary figures and images for: Identification of the powdery mildew resistance gene in wheat breeding line Yannong 99102-06188 via bulked segregant exome capture sequencing
Source: Front Plant Sci. 2022 Sep 6;13:1005627. doi: 10.3389/fpls.2022.1005627 (PMC9489141; doi:10.3389/fpls.2022.1005627)

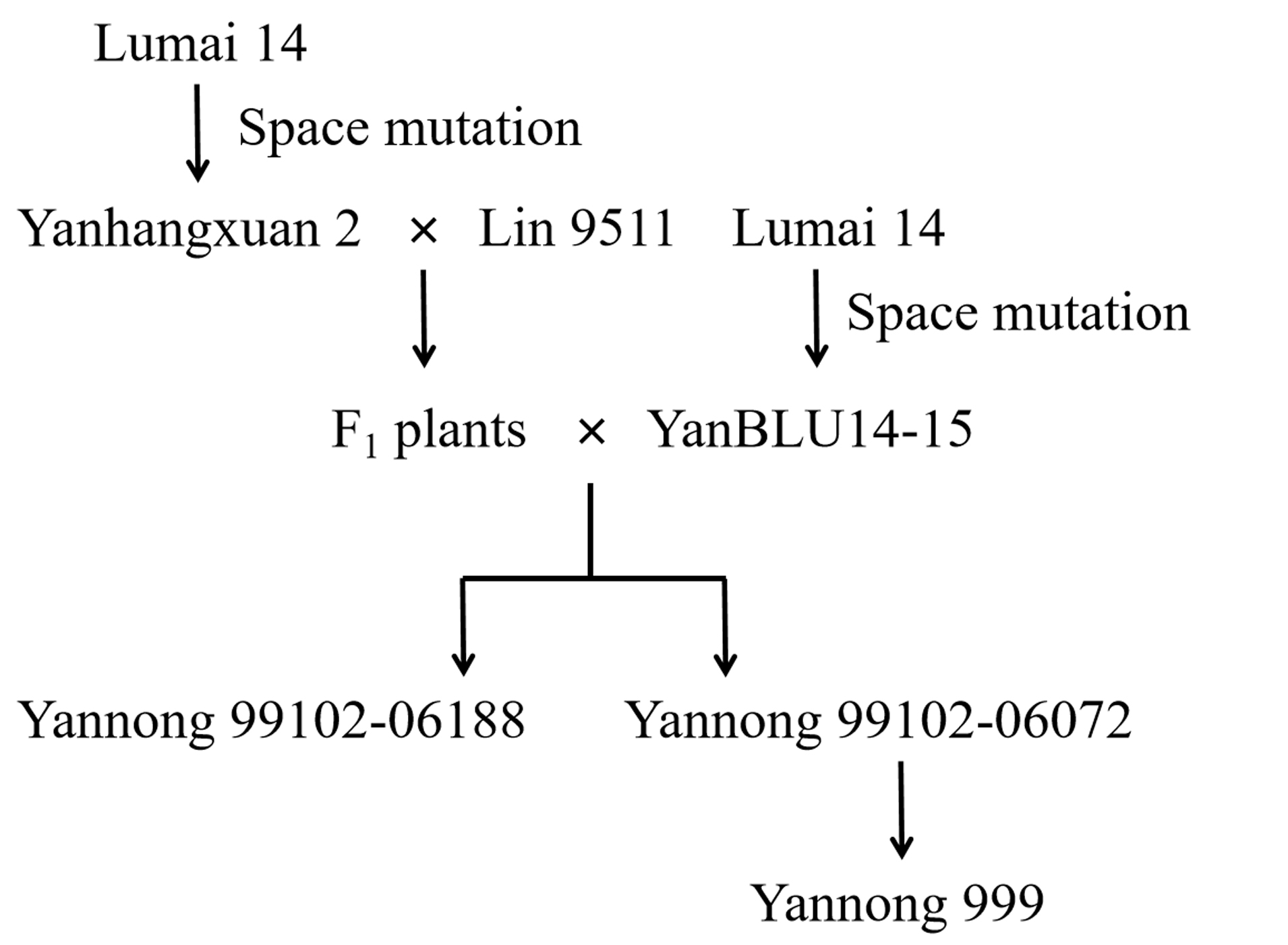

Supplement: Supplementary Figure 1 — Genealogy of the wheat breeding line Yannong 99102-06188 and its relationship with the wheat cultivar Yannong 999. [file Image_1.JPEG]
